# Graph-to-signal transformation based classification of functional connectivity brain networks

**DOI:** 10.1371/journal.pone.0212470

**Published:** 2019-08-22

**Authors:** Tamanna Tabassum Khan Munia, Selin Aviyente

**Affiliations:** Department of Electrical and Computer Engineering, Michigan State University, East Lansing, MI 48824, United States of America; University of Texas at Austin, UNITED STATES

## Abstract

Complex network theory has been successful at unveiling the topology of the brain and showing alterations to the network structure due to brain disease, cognitive function and behavior. Functional connectivity networks (FCNs) represent different brain regions as the nodes and the connectivity between them as the edges of a graph. Graph theoretic measures provide a way to extract features from these networks enabling subsequent characterization and discrimination of networks across conditions. However, these measures are constrained mostly to binary networks and highly dependent on the network size. In this paper, we propose a novel graph-to-signal transform that overcomes these shortcomings to extract features from functional connectivity networks. The proposed transformation is based on classical multidimensional scaling (CMDS) theory and transforms a graph into signals such that the Euclidean distance between the nodes of the network is preserved. In this paper, we propose to use the resistance distance matrix for transforming weighted functional connectivity networks into signals. Our results illustrate how well-known network structures transform into distinct signals using the proposed graph-to-signal transformation. We then compute well-known signal features on the extracted graph signals to discriminate between FCNs constructed across different experimental conditions. Based on our results, the signals obtained from the graph-to-signal transformation allow for the characterization of functional connectivity networks, and the corresponding features are more discriminative compared to graph theoretic measures.

## Introduction

The human brain is a highly interconnected network. While early studies of neurophysiological and neuroimaging data focused on the analysis of isolated regions, i.e. univariate analysis, most of the recent work indicates that the network organization of the brain fundamentally shapes its function [1]. Complex network theory has contributed significantly to the characterization of the topology of FCNs, in particular in the assessment of functional integration and segregation [2, 3]. Thus, generating comprehensive maps of brain connectivity, also known as connectomes, and characterizing these networks has become a major goal of neuroscience [4, 5]. There has been a lot of recent work on classifying different disorders using FCNs as the feature vectors [6, 7]. In this line of work, first a lower dimensional discriminative feature vector is extracted from FCNs using different metrics, then these low-dimensional vectors are fed into a classifier [7]. The major disadvantages of this approach are that original FCNs may be noisy and contain redundant information, and the dimensionality reduction methods are usually supervised which requires the availability of a large number of samples for each class for reliable feature selection.

Recently, this problem has been addressed by using graph theoretic measures which do not depend on the size of the training data set. Specifically, graph theoretic measures such as the path length and clustering coefficient have helped to characterize small-world brain networks [8–10], and the degree distribution has been utilized to characterize scale-free networks [11]. Over the last decade, the study of FCNs through complex network theory has provided new means for discriminating between different neural dysfunctions such as epilepsy [12, 13], depression [14, 15], Alzheimer’s Disease [16, 17], and Parkinson’s Disease [18].

Although graph theoretical approaches provide an elegant way to describe the topology of functional brain networks, these measures suffer from several major shortcomings. First, most network measures are optimally suited for sparse and binary networks. Early work in the area of graph theoretic measures focused on binary networks, leading to the thresholding of networks constructed from neuroimaging studies. However, thresholding poses the problem of over-simplifying FCNs and more importantly, there is no generally accepted criterion to select the threshold [10, 19]. Moreover, the size and density of the thresholded network varies based on the chosen threshold value [2]. Recent studies show that the significance of the difference between groups is strongly dependent on the threshold parameter, i.e. the power of the statistical analysis varies with the threshold [20]. Recently, extensions of graph theoretic measures have been proposed for weighted networks to address some of these issues [21–23]. Second, it has been shown that graph theoretic measures, such as the clustering measure and the small-world parameter, are very sensitive to the size of the network, i.e. the number of nodes, and the density of the connections. Thus, comparing two networks with different edge density may lead to wrong conclusions making it difficult to disentangle experimental effects from those introduced by differences in the average degree [10, 24]. Third, graph theoretic measures are in general non-unique. An example is the small-world measure as two very different network structures may yield similar small-world parameters [25]. Finally, graph theoretic measures do not necessarily reflect the actual mechanism for flow of information in the underlying network, especially for weighted networks such as FCNs. For example, FCNs may not necessarily rely on shortest paths for communication between the nodes, and measures like the characteristic path length and the global efficiency are unable to capture this type of connectivity patterns [2, 26].

In this paper, we propose an alternative approach for feature extraction from FCNs based on graph-to-signal transformation. Unlike graph theoretic measures which often result in a single number, transforming graphs into signals results in as many signals as nodes, and thus can be considered as a lossless transformation. In addition, by transforming graphs into signals it is possible to apply traditional signal processing techniques on the resulting signals in order to extract information from the networks. Two different approaches, i.e. probabilistic [27] and deterministic [28, 29] methods, have been developed to transform networks into signals. The deterministic methods are based on classical multidimensional scaling transforming binary networks into time series [28, 29]. With this transformation, the nodes of the network map into time indices in the resulting signals [28–30].

In this paper, we extend deterministic graph-to-signal transformations from binary to weighted networks using the resistance distance [31]. Some advantages of the resistance distance include invertibility and accounting for the global structure of the graph, thus incorporating information about multiple paths. The resulting signals provide information about the topology of the network which can be used to extract descriptive features from the network. In this paper, we propose to implement well-known signal features such as entropy and statistical moments for these graph-to-signals. The extracted features are naturally low dimensional and are unsupervised, thus do not depend on the quality and the size of the training data unlike FCN based features. Finally, we apply this new transform and the accompanying features to FCNs constructed from an EEG speeded-reaction task experiment. The results obtained from this data set indicate that the proposed graph-to-signal transformation can identify the brain regions central to error-related negativity (ERN). Furthermore, the features extracted from these signals are more discriminative compared to conventional graph theoretic measures and FCN based classification.

## Background

### Phase synchrony

Weighted connectivity networks were constructed from EEG data using a measure of phase synchrony. Each electrode was considered as a vertex of the graph and the weights between vertices were obtained by computing the phase synchrony between two regions. In this paper, the pairwise phase synchrony was computed by using a recently introduced time-frequency phase synchrony (TFPS) measure based on the reduced interference Rihaczek (RID-Rihaczek) time-frequency distribution [32]. For a signal *x*_*i*_(*t*), the RID-Rihaczek distribution is defined as [32]:

$$\begin{array}{c} C_{i}\left(t,f\right)=\int_{-\infty }^{\infty }\int_{-\infty }^{\infty }\exp(-\frac{{\left(\theta \tau \right)}^{2}}{\sigma })\exp(j\frac{\theta \tau }{\sigma })A\left(\theta ,\tau \right)e^{-j(\theta t+2\pi f\tau )}d\tau d\theta , \end{array}$$ (1)

where $\exp(-\frac{{\left(\theta \tau \right)}^{2}}{\sigma })$ is the Choi-Williams kernel, [33], $\exp(j\frac{\theta \tau }{\sigma })$ is the kernel function for the Rihaczek distribution [34] and *A*(*θ*, *τ*) is the ambiguity function of the given signal *x*_*i*_ and is defined as:

$$\begin{array}{c} A\left(\theta ,\tau \right)=\int_{-\infty }^{\infty }x_{i}(u+\frac{\tau }{2})x_{i}^{*}(u-\frac{\tau }{2})e^{j\theta u}du. \end{array}$$ (2)

The instantaneous phase of *x*_*i*_ is computed from *C*_*i*_(*t*, *f*) as:

$$\begin{array}{c} \phi_{i}\left(t,f\right)=\arg[\frac{C_{i}\left(t,f\right)}{|C_{i}\left(t,f\right)|}]. \end{array}$$ (3)

The phase difference between two signal *x*_*i*_ and *x*_*j*_ can then be computed as:

$$\begin{array}{c} \phi_{i,j}\left(t,f\right)=\arg[\frac{C_{i}\left(t,f\right)C_{j}^{*}\left(t,f\right)}{|C_{i}\left(t,f\right)||C_{j}\left(t,f\right)|}]. \end{array}$$ (4)

Phase Locking Value (PLV), which quantifies the phase synchrony between two signals *x*_*i*_ and *x*_*j*_, is defined as the consistency of the phase differences *ϕ*_*i*,*j*_(*t*, *f*) across trials and can be computed as [35]:

$$\begin{array}{c} PLV_{i,j}\left(t,f\right)=\frac{1}{K}\left|\sum_{k=1}^{K}\exp(j\phi_{i,j}^{k}\left(t,f\right))\right|, \end{array}$$ (5)

where *K* is the total number of trials, i.e. the number of times a given stimulus is repeated, and $\phi_{i,j}^{k}\left(t,f\right)$ is the phase difference for the *k*th trial between $x_{i}^{k}$ and $x_{j}^{k}$ as defined by (4). Once the pairwise PLV values are computed between all pairs of electrodes, the weighted adjacency matrix corresponding to the FCN can be constructed as the average of *PLV*_*i*,*j*_(*t*, *f*) within the time interval and frequency band of interest. Thus, the connectivity matrix **W** is constructed such that *W*_*ij*_ = ∑_*t*∈25−75*ms*_∑_*f*∈*θband*_ *PLV*_*i*,*j*_(*t*, *f*), i.e. the average connectivity within 25-75 ms time window and theta (*θ*: 4 − 8*Hz*) frequency band.

### Graph theory

An undirected graph *G* = (*V*, *E*) is defined by a set of *N* nodes, *v*_*i*_ ∈ *V*, and a set of edges, *e*_*ij*_, *i*, *j* ∈ {1, …, *N*}. The relationships between the nodes of the graph is represented by the adjacency matrix **A** = [*A*_*ij*_] for binary graphs, and **W** = [*W*_*ij*_] for weighted graphs. In binary graphs, *A*_*ij*_ = 1 when nodes *i* and *j* are connected and *A*_*ij*_ = 0 when the nodes are not connected. For weighted graphs, *W*_*ij*_ represents the weight of the edge between nodes *i* and *j* and equals to zero when *i* = *j*. The degree matrixΔ is defined as the diagonal matrix with entries $\Delta_{ii}=\sum {}_{\begin{array}{c} j=1\;\\ j\neq i \end{array}}^{N}A_{ij}$, where Δ_*ii*_ is the degree of node *v*_*i*_. Similarly, the degree matrix Δ^*w*^ for weighted networks has diagonal entries $\Delta_{ii}^{w}=\sum_{\begin{array}{c} j=1\;\\ j\neq i \end{array}}^{N}W_{ij}$.

For binary graphs the combinatorial Laplacian **L** is defined as **L** = Δ − **A**. The elements of **L** are:

$$\begin{array}{c} L_{ij}=\{\begin{array}{c} \Delta_{ii},\;\;i=j\;,\\ -1,\;\;(i,j)\in \;E,\\ 0,\;\;otherwise, \end{array} \end{array}$$ (6)

whereΔ_*ii*_ is the degree of node *v*_*i*_. Similarly, the Laplacian for weighted graphs is defined as **L**^*w*^ = Δ^*w*^ − **W**.

### Graph theoretic measures

Complex networks can be characterized using graph theoretic metrics such as the clustering coefficient, characteristic path length, global efficiency, small world parameter and small world propensity [36, 37]. In this paper, we use graph theoretic measures defined for weighted networks as features for classification. Using graph theoretic measures defined for weighted networks circumvents the shortcomings associated with thresholding [2, 19, 20]. The features considered in this paper are as follows.

Clustering coefficient: The mean clustering coefficient is a measure of segregation and reflects mainly the fraction of clustered connectivity available around individual nodes. The clustering coefficient for a weighted network is defined as [38]:

$$\begin{array}{c} C^{w}=\frac{1}{N}\sum_{i\in V}\frac{2t_{i}^{w}}{k_{i}\left(k_{i}-1\right)}, \end{array}$$ (7)

where $t_{i}^{w}$ is the weighted geometric mean of the triangles around a node *i* defined as $t_{i}^{w}=\frac{1}{2}\sum_{j,h\in V}{\left(W_{ij}W_{ih}W_{jh}\right)}^{\frac{1}{3}}$ and *k*_*i*_ is the degree of node *i*.

Characteristic Path Length: The characteristic path length of the network is the average shortest path length between all pairs of nodes in the network. Path length in the brain network represents the potential routes of information flow between two different brain regions and quantifies the potential for functional integration [2]. For a weighted network, the characteristic path length is calculated as [2]:

$$\begin{array}{c} L^{w}=\frac{1}{N}\sum_{i\in V}\frac{\sum_{j\epsilon V,j\neq i}d{}_{ij}^{w}}{\left(N-1\right)}, \end{array}$$ (8)

where $d{}_{ij}^{w}$ is the shortest weighted path length between node *i* and *j* defined as

$$\begin{array}{c} d{}_{ij}^{w}=\sum_{a_{uv}\in g_{i\overset{w}{↔}j}}f(w_{uv}), \end{array}$$ (9)

*f* refers to a map (e.g. an inverse function) from weight to length and $g_{i\overset{w}{↔}j}$ is the shortest weighted path between *i* and *j*.

Global Efficiency: The average inverse shortest path length is defined as the global efficiency of a network. It is a measure of functional integration similar to characteristic path length but can also be computed meaningfully for disconnected networks as an infinite path length results in zero efficiency [39]. The global efficiency for a weighed network is given by [39]:

$$\begin{array}{c} E^{w}=\frac{1}{N}\sum_{i\in V}\frac{\sum_{j\in V,j\neq i}(d{}_{ij}^{w}{)}^{-1}}{\left(N-1\right)}, \end{array}$$ (10)

where $d{}_{ij}^{w}$ is the shortest weighted path length between node *i* and *j* defined by Eq (9).

Small-World Parameter (SW): A network that has significantly more clusters than a random network but approximately the same characteristic path length as a random network is formally defined as a small-world network [40]. Small-world networks are simultaneously strongly clustered and integrated. This phenomenon of small worldness is captured by the small-world parameter which is the ratio of the normalized clustering coefficient to the normalized path length. For a weighted network, the small-world parameter is given as [2, 41]:

$$\begin{array}{c} \sigma^{w}=\frac{C^{w}/C_{rand}^{w}}{L^{w}/L_{rand}^{w}}, \end{array}$$ (11)

where *C* and *C*_*rand*_ are the clustering coefficients of the network and a random network with the same degree distribution, respectively, and *L* and *L*_*rand*_ are the characteristic path lengths of the network and a random network with the same degree distribution, respectively. The random networks are generated using the Erdos-Renyi model with the same number of nodes and connection density.

Small-World Propensity (SWP): Small world propensity is a measure that quantifies the level of small-worldness displayed by a network while accounting for the variation of network density [24]. SWP is measured by computing the deviation of the observed network’s clustering coefficient and characteristic path length from random (*C*_*rand*_, *L*_*rand*_) and lattice (*C*_*lat*_, *L*_*lat*_) networks designed with the same degree distribution and same number of nodes as follows:

$$\begin{array}{c} SWP=1-\sqrt{\frac{\Delta_{C}^{2}+\Delta_{L}^{2}}{2}}, \end{array}$$ (12)

where $\Delta_{C}=\frac{C_{lat}-C}{C_{lat}-C_{rand}}$, and $\Delta_{L}=\frac{L-L_{rand}}{L_{lat}-L_{rand}}$.

In this work, we computed all of these well-known graph theoretic measures and compared them with the graph signal features.

## Methods

### Graph-to-signal transformation based on the resistance distance matrix

The goal of CMDS is to find a projection of the high-dimensional data into a lower dimensional space such that the Euclidean distances between points are preserved [42]. In particular, for our application of transforming graphs into signals, the goal is to obtain coordinate vectors that preserve the functional connectivity between the different brain regions [29].

In order to extract these coordinate vectors, first, the adjacency matrix **A** of a given network is transformed into a squared distance matrix, **D**^(2)^, which is consequently double centered as

$$\begin{array}{c} B=-\frac{1}{2}J_{N}\;D^{\left(2\right)}\;J_{N}, \end{array}$$ (13)

where **D**^(2)^ = **D** ∘ **D** is the entry-wise squared Euclidean distance matrix also known as the Hadamard product, **J**_*N*_ is a centering matrix defined as $J_{N}=I_{N}-\frac{1}{N}1_{N}1_{N}^{T}$, **I**_*N*_ is an *N* × *N* identity matrix, **1**_*N*_ is a *N* × 1 vector of ones, and *T* denotes the transpose. In order to preserve the positive definiteness of **B**, the matrix **D** has to be a valid distance matrix and conditionally negative definite. CMDS has been used in literature for the transformation of binary [28, 43] and weighted networks [44]. For the binary network, the distance **D** is based on the binary adjacency matrix **A**.

In this paper, we propose a graph-to-signal transformation of the weighted graphs using the resistance distance, **R**. The resistance distance was introduced by Klein and Randic as an alternative to the shortest path distance for applications in chemistry [45]. It is inspired by basic circuit theory, where each edge on the graph represents a resistor with value $\frac{1}{W_{ij}}$ [46]. The resistance distance between node *i* and node *j* is defined as *R*_*ij*_, and is computed for complete graphs through the Moore-Penrose pseudo inverse of the Laplacian, **L**, **L**^†^ [28], as

$$\begin{array}{c} R_{ij}=L_{ii}^{†}+L_{jj}^{†}-2L_{ij}^{†}. \end{array}$$ (14)

Each entry *R*_*ij*_ in **R** corresponds to the squared Euclidean distance between nodes *i* and *j* [47]. For a connected graph, *R*_*ij*_ ≤ *d*(*i*, *j*), where *d*(*i*, *j*) is the shortest path distance, and equality condition will hold when there is only one path between *i* and *j* [48]. **R** is a valid squared Euclidean distance matrix as each entry *R*_*ij*_ satisfies the following rules [49]:

$$\begin{array}{ccc} R_{ij} & \geq & 0\;\text{for}\;\text{all}\;\text{i},\;\text{j}\;\text{with}\;\text{equality}\;\text{if}\;\text{and}\;\text{only}\;\text{if}\;\text{i}=\text{j},\\ R_{ij} & = & R_{ji},\\ R_{ij}+R_{jk} & \geq & R_{ik}. \end{array}$$ (15)

As a result, **R** can be directly substituted in (13) to obtain the corresponding Gram matrix **B** as

$$\begin{array}{c} B=-\frac{1}{2}J_{N}\;R\;J_{N}. \end{array}$$ (16)

It can be shown that the resulting matrix **B** is a positive semi-definite matrix with *rank*(**B**) = *C*, *C* ≤ *N*. Therefore, **B** has *C* number of nonzero eigenvalues, and *N* − *C* number of eigenvalues equal to zero.

The next step in graph-to-signal transformation is to perform the spectral factorization of **B**, resulting in $B=P\;\Lambda \;P^{T}=(P\Lambda^{\frac{1}{2}})\times {\left(P\Lambda^{\frac{1}{2}}\right)}^{T}=XX^{T}$, where *Λ* = *diag*(λ_1_, λ_2_, …, λ_*C*_) corresponds to the nonzero eigenvalues of **B**, with λ_1_ ≥ λ_2_ ≥ ⋯ ≥ λ_*C*_, $P\in R^{N\times C}$, and $X\in R^{N\times C}$. Based on **X**, a total of *C* signals of length *N* corresponding to the columns of **X** are obtained. The *i*th signal $x_{i}\in R^{N\times 1}$ is defined as the *i*th column of **X** with *i* = 1, 2, …, *C*. In this paper, we will refer to **x**_*i*_*s* as the signals representing the network.

If the generated signals **X** are not distorted, it is possible to get back to the original network from the signals. First, the resistance distance matrix **R** can be inferred from the graph signals **x**_*i*_s by computing the squared Euclidean distance between the points as follows

$$\begin{array}{c} {\hat{R}}_{ij}=\sum_{c=1}^{C}{\left(x_{c}\left(i\right)-x_{c}\left(j\right)\right)}^{2}, \end{array}$$ (17)

where $\hat{R}$ is the estimated **R**, *C* corresponds to the total number of components and *x*_*c*_(*i*) and *x*_*c*_(*j*) correspond to the *i*th and *j*th entries of the *c*th component. The original adjacency matrix can then be recovered from $\hat{R}$, for both weighted and binary graphs following the procedure detailed in [50].

### Graph signal features

In this section, we describe several well-known features adapted to graph signals. Along with common signal measures like Shannon Entropy (ShEn), skewness and kurtosis, we propose a new measure named graph spectral entropy (GSE) for quantifying the structural information of graphs based on the signals obtained from the networks. The extracted features are explained below.

Shannon Entropy (ShEn): Shannon entropy is a standard entropy measure widely used for signal analysis. It quantifies the order state of a signal through the probability density function of the distribution. Shannon entropy of the *i*th graph signal is computed as [51]:

$$\begin{array}{c} H_{i}=-\sum_{n=1}^{N}Q_{i}\left[n\right]\log_{2}\left(Q_{i}\left[n\right]\right), \end{array}$$ (18)

where *Q*_*i*_ is the probability density function for the *i*th graph signal obtained through the histogram of the signal values. ShEn was computed for each of the graph signals, *x*_*i*_[*n*], *i* = 1, 2, …, *C*, generated through the graph-to-signal transformation. The average entropy, $H=\frac{\sum_{i=1}^{C}H_{i}}{C}$ over all signals was extracted as a feature for consequent analysis.

Skewness and Kurtosis: Skewness (S) [52] and Kurtosis (Ku) [53] of a signal are measures of the third and fourth moments, respectively and are defined as $S_{i}=\frac{\mu_{3i}}{\sigma_{i}}$ and $Ku_{i}=\frac{\mu_{4i}}{\sigma_{i}}$, where *μ*_3*i*_ is the third central moment, *μ*_4*i*_ is the fourth central moment and *σ*_*i*_ is the standard deviation of the *i*th signal. The *nth* central moment can be computed as $\mu_{ni}=\sum_{k=1}^{N}{\left(x_{i}\left[k\right]-\mu_{i}\right)}^{n}Q_{i}\left[k\right]$, where *μ*_*i*_ is the mean of the *i*th signal and *Q*_*i*_ is the probability density function for the *i*th graph signal. The average skewness $S=\frac{\sum_{i=1}^{C}S_{i}}{C}$ and average kurtosis $Ku=\frac{\sum_{i=1}^{C}Ku_{i}}{C}$ measured over all the signals were considered as two features of the graph signal.

Graph Spectral Entropy: We propose a new graph entropy measure based on the spectra of the graph signals. In particular, we propose to compute the graph entropy based on the normalized power spectrum of $x_{i}\left[n\right],\;i=1,2,...,\tilde{C}$, where we consider the $\tilde{C}<C$ signals with highest energy. This parameter is selected empirically similar to the selection of the total number of factors in Principal Components Analysis (PCA). The magnitude spectrum of the *i*th signal is defined as $M_{i}[k]={\left|F\left\{x_{i}\right\}\right|}^{2}$, where F denotes the discrete Fourier transform, $F\left\{x_{i}\right\}=\sum_{n=1}^{N}x_{i}\left[n\right]e^{-\frac{j2\pi nk}{N}}$. The normalized power spectrum of the *i*th signal for the positive frequencies is computed as $P_{i}[k]=\frac{M_{i}[k]}{\sum_{k=0}^{\left\lfloor (N-1)/2\right\rfloor }M_{i}[k]}$, where *k* = 0, 1, …, ⌊(*N* − 1)/2⌋ corresponds to discrete frequency bins [54]. The normalized graph entropy for the *i*th graph signal is defined as

$$\begin{array}{c} H_{i}=-\frac{1}{\log(\lfloor (N-1)/2\rfloor )}\sum_{k=0}^{\left\lfloor (N-1)/2\right\rfloor }P_{i}[k]\log_{2}\left(P_{i}[k]\right), \end{array}$$ (19)

where $i=1,2,...,\tilde{C}$ [54]. Since (19) refers to the Shannon entropy, it is bounded as 0 ≤ *H*_*i*_ ≤ log(*N*^2^/2). We propose to use the normalized power spectrum rather than the original signals for entropy computation since computing the Shannon entropy directly on the signals does not necessarily provide information about the network’s structural content. For example, for a structured network such as the ring network, the corresponding signals are pure sinusoids [28, 55], with almost uniform histograms resulting in high entropy. On the other hand, the power spectrum of a sine wave is well localized at a particular frequency thus its Shannon entropy is theoretically zero. This is consistent with the intuition that a ring network is deterministic and thus, should exhibit low entropy. Thus, the lower bound of *H*_*i*_ is achieved when the distribution is an impulse, and the upper bound occurs when the distribution is uniform. In terms of graph structures, the lower bound corresponds to the ring lattice and the upper bound corresponds to a random network.

In order to account for the variation in the network entropy as the probability of attachment varies, we propose to weigh the entropy of each graph signal using its energy using weights $w_{i}=\frac{∥x_{i}{∥}_{1}}{\sqrt{N}{∥x_{i}∥}_{2}}.\;w_{i}\in \left[\frac{1}{\sqrt{N}},1\right]$, using the fact that ${∥x∥}_{2}\leq {∥x∥}_{1}\leq \sqrt{N}{∥x∥}_{2}$. These weights are normalized across signals as ${\tilde{w}}_{=}\frac{w_{i}}{\sqrt{\tilde{C}}{∥w∥}_{2}}$, where $w=(w_{1},w_{2},...,w_{\tilde{C}})$. We define the weighted graph spectral entropy (GSE) as

$$\begin{array}{c} GSE=\sum_{i=1}^{\tilde{C}}{\tilde{w}}_{i}H_{i}. \end{array}$$ (20)

This definition of network entropy is independent of graph theoretic measures and the eigenspectrum of the adjacency matrix. The structural information of the network is thus obtained from the signals that already contain the network topological information.

### Illustration of the proposed measure

**Fig 1:**
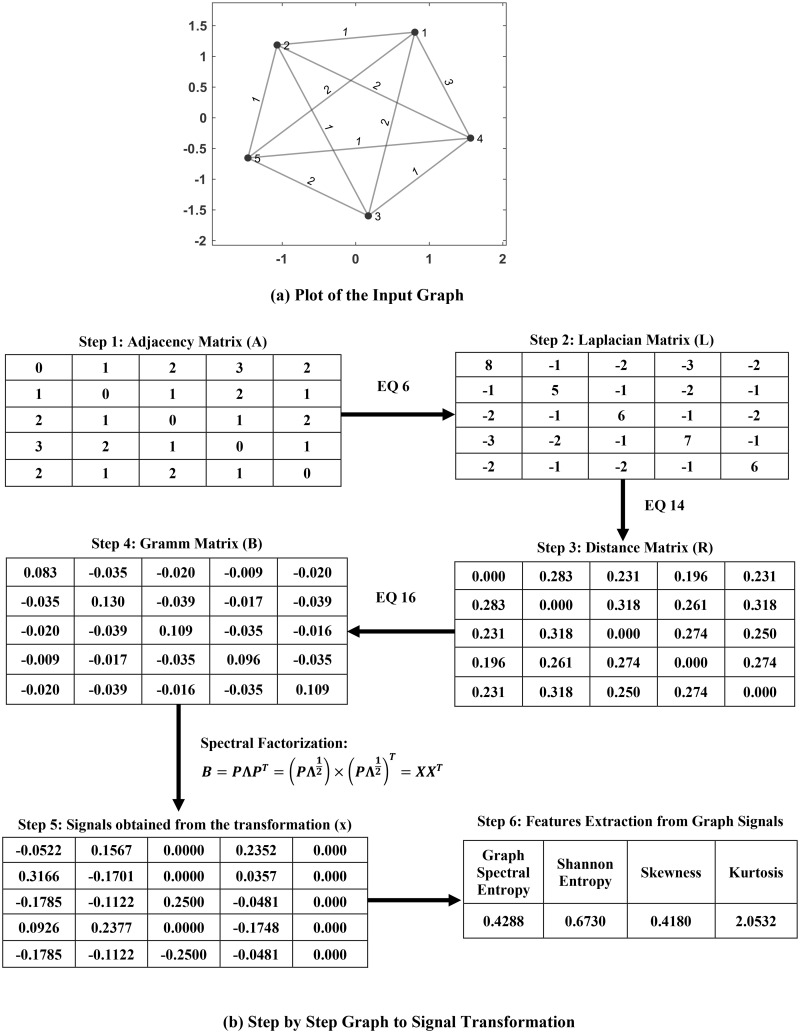
An example of graph to signal transformation and corresponding feature extraction for a 5 × 5 network. (a) Plot of the generated graph; (b) Step by step graph-to-signal transformation for graph features extraction.

Proposed graph to signal transformation is illustrated step by step for a toy example in Fig 1. Here a 5 × 5 weighted network was generated and transformed through proposed graph-to-signal transformation for extracting the graph signal features. Fig 1(a) shows the plot of the generated toy graph. The step-wise signal generation procedure is illustrated in Fig 1(b). The adjacency matrix was generated in the first step followed by the generation of the Laplacian matrix **L** (from (6)). The resistance distance matrix **R** was computed using (14). The Gram matrix **B** was computed from **R** following (16). In step 5, graph signals are obtained through spectral factorization of **B**. As described above, four graph signal features namely graph spectral entropy, Shannon entropy, skewness and kurtosis were extracted from these signals in step 6.

## Results

### Simulations

#### Graph-to-signal transformation for binary networks

**Fig 2:**
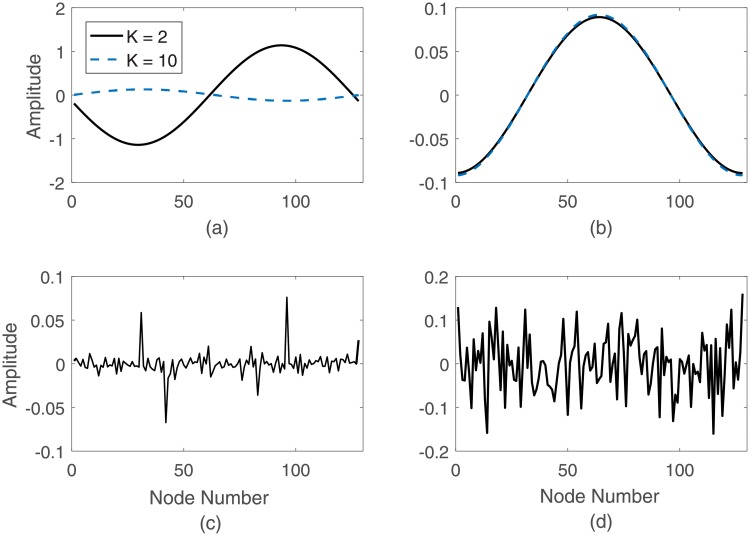
Signal representation of binary network. Top: Signal representation of a k-regular graph with degree *K* = 2 and *K* = 10; a. Resistance distance measure (**R**), b. Distance measure (**D**); Bottom: Signal representation of an Erdős-Rènyi network with probability of attachment *p* = 0.5; c. Resistance distance measure (**R**), d. Distance measure (**D**). For all networks *N* = 128.

We first compare the proposed distance measure, **R**, with respect to **D** for binary networks. For this purpose, we qualitatively compare the signals obtained from multiple binary networks. First, we simulate two k-regular graphs with *N* = 128 nodes and average degrees *K* = 2 and *K* = 10. Fig 2(a) and 2(b) show the graph signals with the highest eigenvalue obtained from **R** and the distance **D**, respectively. As expected, the signals based on the resistance distance matrix are sinusoidal signals (Fig 2(a)). From these figures, it is observed that the amplitude of signals obtained from **R** is inversely proportional to the average degree, *K*, yielding a higher amplitude when *K* = 2 and a smaller amplitude when *K* = 10. On the other hand, **D** cannot distinguish between k-regular graphs with varying average degrees.

We also compared both methods for an Erdős-Rènyi binary graph for a probability of attachment *p* equal to 0.5. For the original distance matrix **D**, the signals are random signals (Fig 2(d)), as previously shown in [56]. On the other hand, signals estimated from **R** still exhibit a random structure, with peaks that are inversely proportional to *p* (Fig 2(c)). The location of these peaks corresponds to the nodes with the smallest degree, i.e. the largest peak occurs in the first signal and corresponds to the node with the smallest degree. For the resistance distance, a node with small degree will have a high resistance distance between it and the remaining nodes in the network. Therefore, signals obtained from the transformation of binary networks through the resistance distance are more informative than those obtained from **D**.

#### Graph-to-signal transformation for weighted graphs

**Fig 3:**
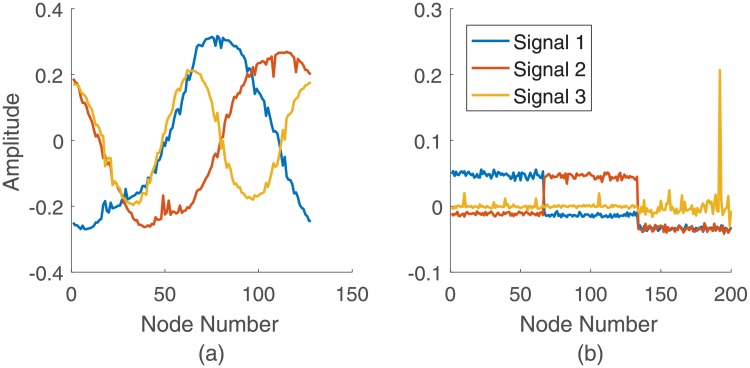
Signal representation of weighted network. First three signals from a. A weighted small-world network with *K* = 6, and *N* = 128 nodes; b. A weighted stochastic block network with probability of attachment *p* = 0.3, and *N* = 200 nodes.

The proposed transformation was also assessed on weighted networks. Fig 3(a) shows the signals resulting from a small-world network with average degree *K* = 6, and *N* = 128 nodes. As seen in Fig 3(a), for a network with a low rewiring probability, *p* = 0.1, the resulting signals are sinusoidal signals with some noise. This is consistent with previous work on binary networks [28], where it has been shown that the small-world network is equivalent to a k-regular graph network plus noise.

In addition to the small-world network, we investigated the graph-to-signal transformation of a weighted stochastic block network consisting of 200 nodes and with fixed probability of attachment, *p* = 0.3 and 3 clusters (Fig 3(b)). The weighted stochastic block network generalizes the stochastic block model to networks with edge weights drawn from any exponential family distribution [57]. Using this model, each node *i* belongs to one of *K* blocks or communities. *i*, and each edge *A*_*ij*_ exists with a probability _*ij*_ that depends only on the group memberships of the connecting vertices. Nodes in the same block are stochastically equivalent, indicating their equivalent roles in generating the network’s structure. The stochastic block model is fully specified by a vector *z* denoting the group membership of each vertex and a *K* × *K* matrix of cluster connection probabilities. Weighted stochastic block model extends this by allowing the edges to have some weight values. The weights are assigned randomly from the uniform distribution in the interval [0, 1]. It can be observed from these figures that the first *K* − 1 signals reveal the clusters’ structure, and the *K*^*th*^ signal is an impulse. In addition, the size of each cluster can be inferred from the support of the constant regions in the first *K* − 1 signals. Thus, the proposed approach effectively transforms weighted networks into signals and reflect structural properties of the networks.

### EEG data

In this paper, we analyze an EEG dataset from a previously published cognitive control-related error processing study [58]. The study was designed following the experimental protocol approved by the Institutional Review Board (IRB) of the Michigan State University. The data collection was performed in accordance with the guidelines and regulation established by this protocol. Written and informed consent was collected from each participant before data collection.

**Fig 4:**
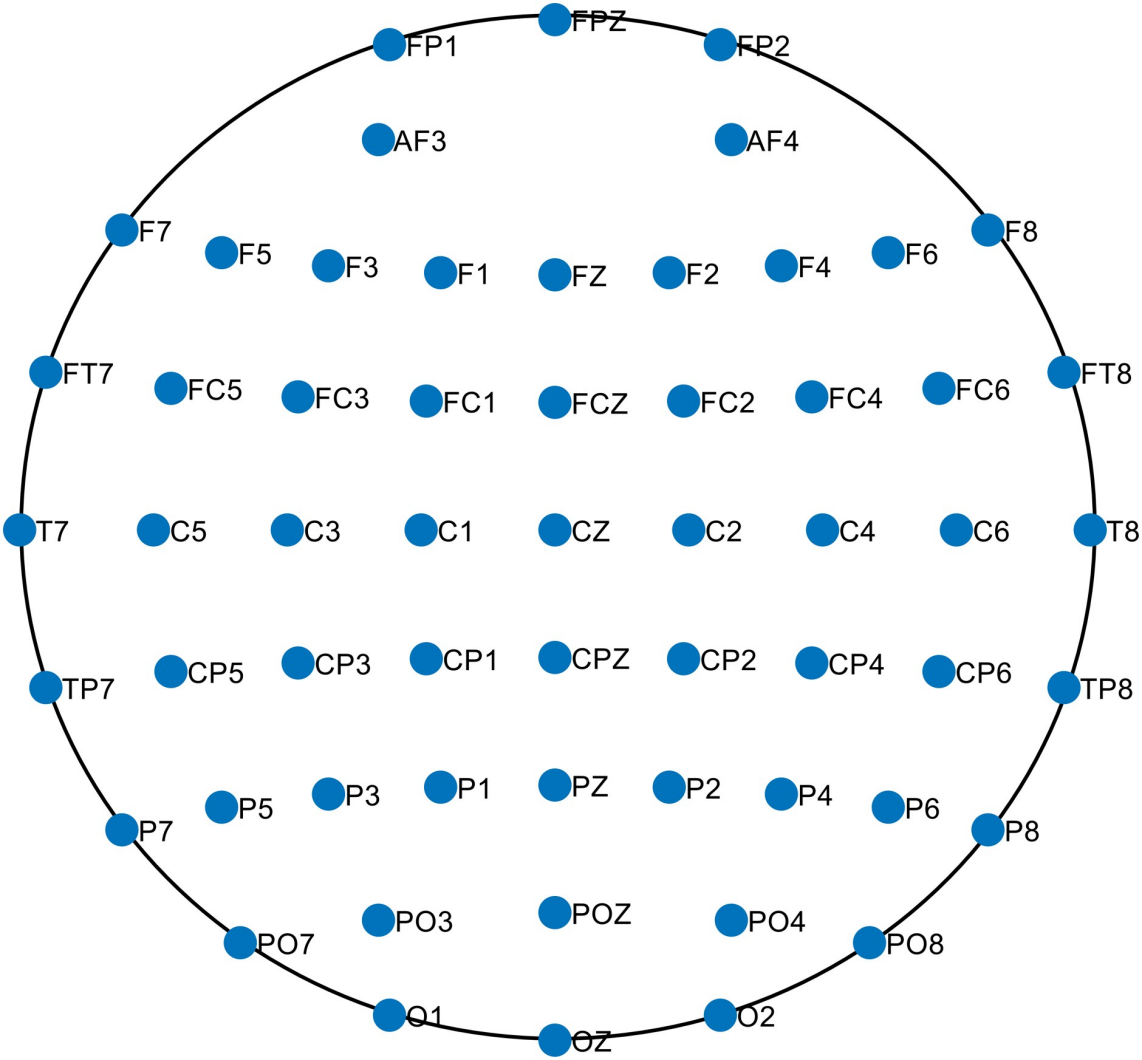
EEG channel locations used for constructing the FCNs.

The experiment consisted of a speeded-reaction Flanker task [59] in which subjects identified the middle letter on a five-letter string, being congruent (e.g. MMMMM) or incongruent (e.g. MMNMM) with respect to the Flanker letters. Flanker letters (e.g. MM MM) were shown during the first 35 ms of each trial, and during the following 100 ms the Flanker and target letters were shown on the screen. This was followed by an inter-trial interval of variable duration ranging from 1200 ms to 1700 ms. A total of 6 blocks consisting of 80 trials composed the experiment, and letters were changed between blocks. EEG responses were recorded by the 64 electrode ActiveTwo system (BioSemi, Amsterdam, The Netherlands). The sampling frequency was 512 Hz. The EEG channel locations are given in Fig 4.

**Fig 5:**
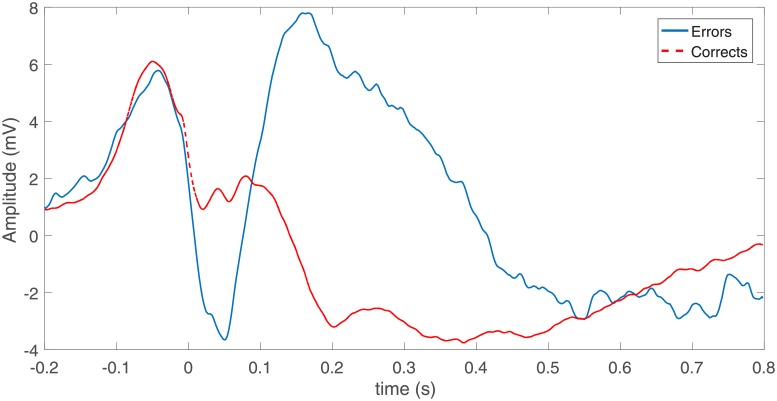
Average error and correct responses at FCz electrode across all trials and all subjects.

Trials containing artifacts were rejected and volume conduction was reduced through the Current Source Density (CSD) Toolbox [60]. A total of 18 subjects and 58 channels were considered for the analysis, for which the total number of error trials ranged from 20 to 61. The same number of correct responses was chosen randomly. Fig 5 shows the event-related potentials for error and correct responses, i.e. error-related negativity (ERN) and correct-related negativity (CRN), from electrode FCz averaged over trials and subjects. As can be seen from this figure, ERN has a larger negative amplitude with the peak within 0-100 ms, where 0 refers to the response time.

In this paper, we are interested in studying the differences in the FCNs corresponding to error-related negativity (ERN) and the correct-related negativity (CRN) through a classification task. Previous studies have shown that the ERN is associated with increased synchronization in the theta band (4-8 Hz) between electrodes in the central and lateral frontal regions [58, 61, 62]. For this reason, a FCN was constructed for each subject by averaging the PLV over the time window 25-75 ms and the frequency bins corresponding to the theta band per subject and response type. This results in two FCNs of size 58 × 58 per subject, one corresponding to error responses and the other to correct responses. A total of 58 signals are extracted from the graph-to-signal transformation of each FCN. The mean ± standard deviation of stress function for the two response types are 4.19^−19^ ± 1.12^−18^ (CRN) and 3.70^−19^ ± 1.16^−18^ (ERN).

#### Graph-to-signal transformation of FCNs

**Fig 6:**
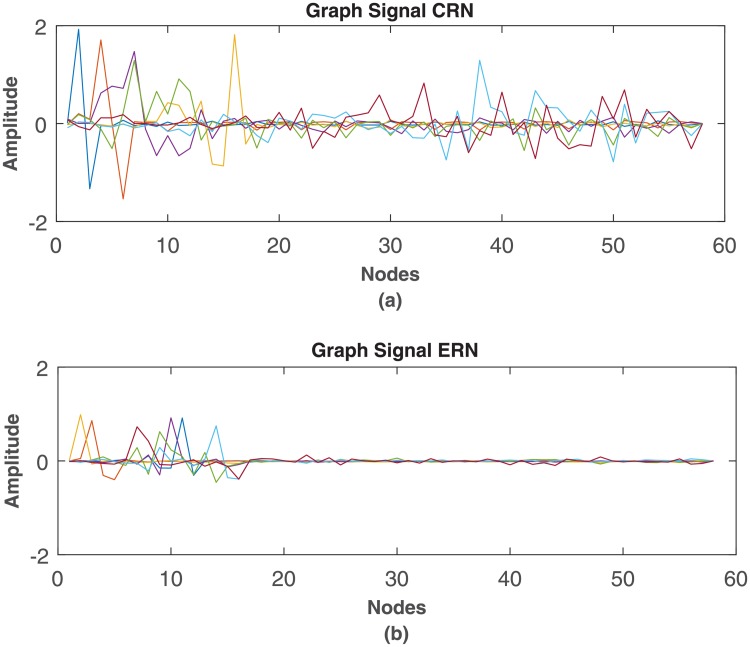
Graph signal representation. The first six signals obtained from graph-to-signal transformation of a. CRN networks; b. ERN networks.

FCNs constructed for ERN and CRN responses were first averaged to obtain representative networks and then transformed into signals using (16). For illustration purposes, we show the first six graph signals corresponding to the correct and the error responses in Fig 6(a) and 6(b), respectively. We focus on the first six signals obtained from this transformation as the eigenvalues of the matrix **B** in (16) drop off significantly after the sixth eigenvalue. As the graph signals are a function of the nodes or different electrodes, the location of the peaks of the graph signals signify the distribution of spatial activity. It can be observed from Fig 6 that while the energy of the graph signals from CRN is distributed uniformly across the 58 brain regions, the energies of the ERN graph signals are more concentrated within the first 20 electrodes, which correspond to the frontal and frontal-central regions. This implies that right after an error response most of the brain activity centralizes within the frontal regions. This is line with prior work indicating the role of prefrontal cortex during ERN [62, 63].

**Fig 7:**
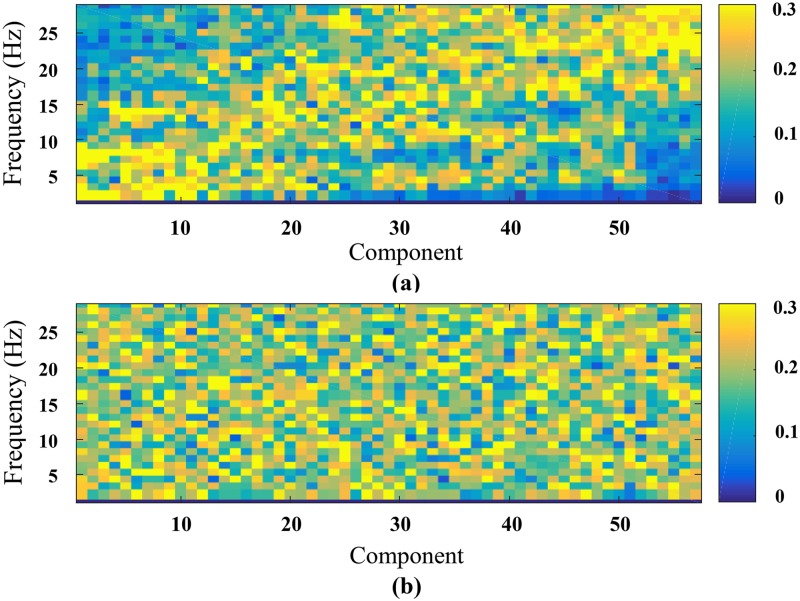
Magnitude spectrum representation. Magnitude Spectrum for each signal obtained through graph-to-signal transformation for a. Error responses; b. Correct responses. The spectrum of error response suggests an organised structure as the frequency content of the signals increases with the signal number whereas the spectrum for the correct responses suggest a random network structure.

Fig 7(a) and 7(b) show the magnitude spectra of the signals corresponding to average error and correct FCNs. For the average FCN constructed from error responses, the frequency content of the signals increases with the signal number, suggesting an organized structure such as k-regular graph networks. On the other hand, the spectra of graph signals corresponding to correct responses suggest a random network structure.

#### Feature extraction

For both ERN and CRN networks, graph theoretic and graph signal features were extracted for each network constructed for each subject and response type, i.e. a total of 36 networks. A total of 5 graph theoretic features (clustering coefficient, characteristic path length; global efficiency; small world parameter and small world propensity) were extracted for each network corresponding to each subject resulting in a feature matrix of dimension 36 × 5. On the other hand, for graph signals, four features named graph spectral entropy, skewness, kurtosis and Shannon entropy were extracted for each signal and then averaged across the graph signals corresponding to each network resulting in a feature matrix of dimension 36 × 4.

#### Classification of FCNs

In this section, we evaluate the classification power of the features extracted from graph signals and compare these features with conventional graph theoretic measures as well as the full FCNs used as feature vectors. For a comprehensive comparison, we employed a set of classifiers including support vector machines (SVM), linear discriminant analysis (LDA), logistic regression and k-nearest neighbor (kNN) (with *k* = 20).

As we have a small dataset (n = 18), the accuracy of each classification method was determined based on its prediction accuracy on leave-one-out prediction technique. Leave-one-out method for validation is a particular case of cross-validation where all test subsets comprise of a single instance. As reported by Kotsiantis *et al*., this type of validation considers all the instances and computationally more expensive, but is beneficial when the most accurate approximation of a classifier’s performance is required [64]. Though this method is computationally expensive, we have used this method to ensure the most accurate estimate of the classifier’s error rate.

Since the operation involves binary classification, sensitivity and specificity defined as follows were used as performance measures in addition to accuracy.

$$\begin{array}{c} Sensitivity=\frac{TP}{TP+FN}, \end{array}$$ (21)

where TP = True Positive; FN = False Negative; and

$$\begin{array}{c} Specificity=\frac{TN}{TN+FP}, \end{array}$$ (22)

where, TN = True Negative and FP = False Positive.

**Fig 8:**
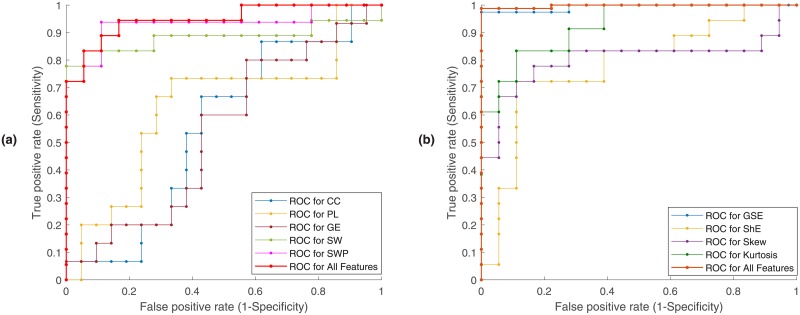
ROC curves for all features. a. Graph Theoretic Features; b. Graph Signal Features.

In order to determine which measure, as a continuous test statistics, best discriminates between error and correct networks, we also computed receiver operating characteristic (ROC) curve for each measure as shown in Fig 8. In the ROC curve, the sensitivity or true positive rate is plotted as a function of the 1-Specificity or false positive rate for different threshold values. As a result, each point of the ROC plot represents a sensitivity/specificity pair that corresponds to a decision threshold. The overall accuracy of the test point can be detected from the proximity of its ROC plot to the upper left corner [57]. For our experiment, the threshold values were computed through threshold averaging method [65]. For each ROC curve, the area under the curve (AUC) was also computed as it serves as a quantitative measure of the discrimination power of the test statistics.

Table 1 shows the classification performance for full network as feature matrix along with graph theoretic (clustering coefficient, path length, global efficiency, small world and small world propensity) and graph signal (proposed graph spectral entropy, Shannon entropy, skewness and kurtosis) features in terms of accuracy, sensitivity, specificity and AUC for different classifiers. From these results, it can be seen that the classification accuracy is much lower when the FCNs are used as features. This is due to the fact that FCNs may be noisy making it hard to discriminate between the two response types. For the graph theoretic features, the small world propensity is the most effective feature. An overall accuracy of 94.4% was obtained by linear SVM using all the graph theoretic features. Moreover, the FCNs constructed from error responses exhibited significantly increased small-world (*p* = 0.00203, Wilcoxon rank-sum test with *p* < 0.01) and small-world propensity (*p* = 0.0008280, Wilcoxon rank-sum test with *p* < 0.01) measure compared to the FCNs from correct responses. This finding of decreased small-world characteristics in correct response networks is indicative of increased randomness and is in line with previous studies that reported increased small-worldness for ERN compared to CRN [23]. For the graph signal features, it can be seen that the graph spectral entropy was the most effective graph signal feature. An overall accuracy of 97.2% was obtained by linear SVM using all features for discriminating between ERN and CRN connectivity networks. Along with the overall 3% improvement of accuracy, the AUC also increased from 0.95 to 0.99 compared to the graph theoretic measures. Moreover, FCNs from correct responses show higher entropy than FCNs from error responses and this difference is significant (*p* = 0.0000554, Wilcoxon rank-sum test with *p* < 0.01). This is consistent with the fact that the error-related negativity is associated with increased synchronization which results in less random networks and hence lower network entropy.

**Table 1:** Classification of ERN and CRN functional connectivity networks using graph theoretic and graph signal features.

Features	Linear SVM	LDA	Logistic Regression	KNN
FCN	58.30% (Se:50%, Sp:67%, AUC:0.52)	55.6% (Se:61%, Sp:50%, AUC:0.5)	69.40% (Se:72%, Sp:67%, AUC:0.67)	61.10% (Se:61%, Sp:33%, AUC:0.46)
CC	58.30% (Se:61%, Sp:56%, AUC:0.60)	44.40% (Se:44%, Sp:44%, AUC:0.56)	52.80% (Se:50%, Sp:56%, AUC:0.56)	47.30% (Se:33%, Sp:61%, AUC:0.47)
PL	61.10% (Se:56%, Sp:67%, AUC:0.60)	52.80% (Se:50%, Sp:56%, AUC:0.55)	55.60% (Se:44%, Sp:67%, AUC:0.48)	38.90% (Se:44%, Sp:33%, AUC:0.46)
GE	55.60% (Se:47%, Sp:64%, AUC:0.64)	52.80% (Se:39%, Sp:67%, AUC:0.63)	50.10% (Se:44%, Sp:56%, AUC:0.55)	44.40% (Se:61%, Sp:28%, AUC:0.59)
SW	81.70% (Se:84%, Sp:79%, AUC:0.86)	78.90% (Se:79%, Sp:79%, AUC:0.71)	76.10% (Se:79%, Sp:73%, AUC:0.71)	81.70% (Se:96%, Sp:85%, AUC:0.81)
SWP	84.20% (Se:88%, Sp:81%, AUC:0.94)	80.10% (Se:82%, Sp:78%, AUC:0.77)	77.20% (Se:80%, Sp:74%, AUC:0.73)	80.40% (Se:86%, Sp:75%, AUC:0.83)
All GTF	94.40% (Se:100%, Sp:89%, AUC:0.95)	91.70% (Se:89%, Sp:94%, AUC:0.88)	88.90% (Se:83%, Sp:94%, 0.56, AUC:0.92)	94.10% (Se:99%, Sp:89%, AUC:0.86)
GSE	85.90% (Se:88%, Sp:83%, AUC:0.97)	71.40% (Se:69%, Sp:72%, AUC:0.97)	81.50% (Se:80%, Sp:83%, AUC:0.95)	76.10% (Se:80%, Sp:72%, AUC:0.94)
ShE	66.70% (Se:89%, Sp:56%, AUC:0.76)	63.90% (Se:85%, Sp:43%, AUC:0.77)	77.80% (Se:89%, Sp:67%, AUC:0.76)	61.10% (Se:61%, Sp:61%, AUC:0.58)
S	77.20% (Se:80%, Sp:74%, AUC:0.83)	71.70% (Se:83%, Sp:68%, AUC:0.80)	74.50% (Se:72%, Sp:77%, AUC:0.80)	44.40% (Se:56%, Sp:33%, AUC:0.61)
Ku	80.60% (Se:78%, Sp:83%, AUC:0.83)	80.60% (Se:94%, Sp:67%, AUC:0.83)	77.80% (Se:72%, Sp:83%, AUC:0.81)	75.00% (Se:78%, Sp:72%, AUC:0.72)
All GSF	97.20% (Se:100%, Sp:94%, AUC:0.99)	97.20% (Se:100%, Sp:94%, AUC:0.99)	94.50% (Se:100%, Sp:90%, AUC:0.94)	94.20% (Se:94%, Sp:94%, AUC:0.97)

Se: Sensitivity; Sp: Specificity; AUC: Area Under the Curve; CC: Clustering Coefficient; PL: Characteristic Path Length; GE: Global Efficiency; SW: Small World Parameter; SWP: Small World Propensity; All GTF: All Graph Theoretic Features; GSE: Graph Spectral Entropy; ShE: Shannon Entropy; S: Skewness; Ku: Kurtosis; All GSF: All Graph Signal Features.

Comparing the graph theoretic and graph signal features in Table 1, we can see the graph spectral entropy has the highest AUC, indicating that among all features, graph spectral entropy is the most effective test statistic to discriminate between the two response types. Therefore, graph spectral entropy is more sensitive to the structural changes in the network compared to small-world and small-world propensity measures in this study.

In order to illustrate the differences between weighted FCNs vs. thresholded binary FCNs for classification, proposed analysis was performed for thresholded FCNs as well. We generated thresholded FCNs using the data driven orthogonal minimal spanning trees (OMSTs) approach described in [66]. For the binary FCNs, we used the distance matrix **D** to be the original adjacency matrix whereas we use the resistance matrix **R** for the weighted FCNs. The same features were extracted for the graph signals for both binary and weighted FCNs. The results are given in Table 2. As it can be seen from this Table 2, the graph signal features are more discriminative for weighted FCNs compared to the binary ones with a difference in accuracy around 6% and AUC of 0.91 vs. 0.88. This is due to the fact that some information is lost through the process of thresholding.

**Table 2:** Classification of thresholded binary ERN and CRN functional connectivity networks using all graph theoretic features (GTF) and graph signal features (gsf).

Features	Linear SVM	LDA	Logistic Regression	KNN
All Thresholded GTF	83.30% (Se:89%, Sp:78%, AUC:0.88)	77.80% (Se:78%, Sp:78%, AUC:0.84)	80.00% (Se:78%, Sp:83%, AUC:0.85)	69.40% (Se:78%, Sp:61%, AUC:0.69)
All Thresholded GSF	88.90% (Se:94%, Sp:83%, AUC:0.91)	83.80% (Se:90%, Sp:77%, AUC:0.81)	86.10% (Se:94%, Sp:78%, AUC:0.87)	75.00% (Se:80%, Sp:70%, AUC:0.85)

GTF: Graph Theoretic Features; GSF:Graph Signal Features; Se: Sensitivity; Sp: Specificity; AUC: Area Under the Curve.

Although the proposed approach has several merits as illustrated in this paper, the analysis is limited to a single data set with a small sample size. As such, the methodology proposed here can be used to guide similar studies with larger sample sizes so that more rigorous quantitative and qualitative analysis can be performed. It is important to note that the major novelty of the current paper is the introduction of a new framework to analyze FCNs rather than the introduction of new feature extraction and classification methods. With graph theoretic metrics, the whole network is reduced to a single number, e.g. small-world parameter. Although this may be attractive for purposes of data reduction and summarizing network topology, this approach also results in some loss of information. Graph-to-signal transformation, on the other hand, results in as many vectors or signals as the number of nodes in the network. Moreover, it is possible to reconstruct the network from these signals unlike graph theoretic metrics. Therefore, graph theoretic metrics can be thought of as lossy compression applied on the network whereas graph-to-signal transformation is a lossless operation. Any well-known signal processing algorithm and feature extraction method can be easily applied to these graph signals. Consequently, future work could explore extracting different types of features like energy, bandwidth, spectral features and other features like Hurst exponent, Lyapunov exponent, Hjorth parameters, correlation coefficients etc. [67] from signals obtained by the proposed graph-to-signal transformation. Exploration of different features may lead to the interpretation of more subtle characteristics of the complex networks which is not possible using the conventional graph theoretic features.

## Conclusion

In this paper, we introduced a new graph-to-signal transformation for weighted FCNs. The signals obtained from this transformation were used to characterize the networks and to extract discriminative features. Results acquired from this study indicate that the features extracted from graph signals are more discriminative compared to conventional graph theoretic measures and the original FCNs for classifying between error and correct responses. In particular, the graph spectral entropy decreases during the ERN interval, while the entropy increases after correct responses. This implies that ERN has a more modular structure implying increased segregation. This finding is in line with previous research showing more localized activity during ERN compared to CRN [68]. Therefore, accumulated evidence from this study suggests that the proposed graph-to-signal transformation based approach can be used to successfully characterize the dynamics of the functional connectivity networks.

## References

[1] Bullmore E, Sporns O. Complex brain networks: graph theoretical analysis of structural and functional systems. Nature Reviews Neuroscience. 2009;10(3):186–198. 10.1038/nrn2575 19190637

[2] Rubinov M, Sporns O. Complex network measures of brain connectivity: uses and interpretations. Neuroimage. 2010;52(3):1059–1069. 10.1016/j.neuroimage.2009.10.003 19819337

[3] Fallani FDV, Richiardi J, Chavez M, Achard S. Graph analysis of functional brain networks: practical issues in translational neuroscience. Phil Trans R Soc B. 2014;369(1653):20130521 10.1098/rstb.2013.0521 25180301 PMC4150298

[4] Van Essen DC, Ugurbil K, Auerbach E, Barch D, Behrens T, Bucholz R, et al The Human Connectome Project: a data acquisition perspective. Neuroimage. 2012;62(4):2222–2231. 10.1016/j.neuroimage.2012.02.018 22366334 PMC3606888

[5] Kandel ER, Markram H, Matthews PM, Yuste R, Koch C. Neuroscience thinks big (and collaboratively). Nature Reviews Neuroscience. 2013;14(9):659 10.1038/nrn3578 23958663

[6] Anderson JS, Nielsen JA, Froehlich AL, DuBray MB, Druzgal TJ, Cariello AN, et al Functional connectivity magnetic resonance imaging classification of autism. Brain. 2011;134(12):3742–3754. 10.1093/brain/awr263 22006979 PMC3235557

[7] Du Y, Fu Z, Calhoun VD. Classification and prediction of brain disorders using functional connectivity: promising but challenging. Frontiers in neuroscience. 2018;12 10.3389/fnins.2018.00525 PMC6088208 30127711

[8] Telesford QK, Joyce KE, Hayasaka S, Burdette JH, Laurienti PJ. The ubiquity of small-world networks. Brain connectivity. 2011;1(5):367–375. 10.1089/brain.2011.0038 22432451 PMC3604768

[9] Bassett DS, Bullmore E. Small-world brain networks. The neuroscientist. 2006;12(6):512–523. 10.1177/1073858406293182 17079517

[10] Bassett DS, Bullmore ET. Small-world brain networks revisited. The Neuroscientist. 2017;23(5):499–516. 10.1177/1073858416667720 27655008 PMC5603984

[11] Albert R, Barabási AL. Statistical mechanics of complex networks. Reviews of modern physics. 2002;74(1):47 10.1103/RevModPhys.74.47

[12] Rajpoot K, Riaz A, Majeed W, Rajpoot N. Functional connectivity alterations in epilepsy from resting-state functional MRI. PloS one. 2015;10(8):e0134944 10.1371/journal.pone.0134944 26252668 PMC4529140

[13] Centeno M, Carmichael DW. Network connectivity in epilepsy: resting state fMRI and EEG–fMRI contributions. Frontiers in neurology. 2014;5:93 10.3389/fneur.2014.00093 25071695 PMC4081640

[14] Zeng LL, Shen H, Liu L, Wang L, Li B, Fang P, et al Identifying major depression using whole-brain functional connectivity: a multivariate pattern analysis. Brain. 2012;135(5):1498–1507. 10.1093/brain/aws059 22418737

[15] Kaiser RH, Whitfield-Gabrieli S, Dillon DG, Goer F, Beltzer M, Minkel J, et al Dynamic resting-state functional connectivity in major depression. Neuropsychopharmacology. 2015;. 10.1038/npp.2015.352 PMC4869051 26632990

[16] Sheline YI, Raichle ME. Resting state functional connectivity in preclinical Alzheimer’s disease. Biological psychiatry. 2013;74(5):340–347. 10.1016/j.biopsych.2012.11.028 23290495 PMC3537262

[17] Wang K, Liang M, Wang L, Tian L, Zhang X, Li K, et al Altered functional connectivity in early Alzheimer’s disease: a resting-state fMRI study. Human brain mapping. 2007;28(10):967–978. 10.1002/hbm.20324 17133390 PMC6871392

[18] Sang L, Zhang J, Wang L, Zhang J, Zhang Y, Li P, et al Alteration of brain functional networks in early-stage Parkinson’s disease: A resting-state fmri study. PloS one. 2015;10(10):e0141815 10.1371/journal.pone.0141815 26517128 PMC4627652

[19] Lee H, Kang H, Chung MK, Kim BN, Lee DS. Persistent brain network homology from the perspective of dendrogram. IEEE transactions on medical imaging. 2012;31(12):2267–2277. 10.1109/TMI.2012.2219590 23008247

[20] Langer N, Pedroni A, Jäncke L. The problem of thresholding in small-world network analysis. PloS one. 2013;8(1):e53199 10.1371/journal.pone.0053199 23301043 PMC3536769

[21] Bassett DS, Wymbs NF, Porter MA, Mucha PJ, Carlson JM, Grafton ST. Dynamic reconfiguration of human brain networks during learning. Proceedings of the National Academy of Sciences. 2011;. 10.1073/pnas.1018985108 PMC3088578 21502525

[22] Rubinov M, Sporns O. Weight-conserving characterization of complex functional brain networks. Neuroimage. 2011;56(4):2068–2079. 10.1016/j.neuroimage.2011.03.069 21459148

[23] Bolanos M, Bernat EM, He B, Aviyente S. A weighted small world network measure for assessing functional connectivity. Journal of neuroscience methods. 2013;212(1):133–142. 10.1016/j.jneumeth.2012.10.004 23085279

[24] Muldoon SF, Bridgeford EW, Bassett DS. Small-world propensity and weighted brain networks. Scientific reports. 2016;6:22057 10.1038/srep22057 26912196 PMC4766852

[25] Papo D, Zanin M, Pineda-Pardo JA, Boccaletti S, Buldú JM. Functional brain networks: great expectations, hard times and the big leap forward. Philosophical Transactions of the Royal Society of London B: Biological Sciences. 2014;369(1653):20130525 10.1098/rstb.2013.0525 25180303 PMC4150300

[26] Estrada E, Hatano N. Communicability in complex networks. Physical Review E. 2008;77(3):036111 10.1103/PhysRevE.77.036111 18517465

[27] Weng T, Zhao Y, Small M, Huang DD. Time-series analysis of networks: Exploring the structure with random walks. Physical Review E. 2014;90(2):022804 10.1103/PhysRevE.90.022804 25215778

[28] Shimada Y, Ikeguchi T, Shigehara T. From networks to time series. Physical review letters. 2012;109(15):158701 10.1103/PhysRevLett.109.158701 23102373

[29] Haraguchi Y, Shimada Y, Ikeguchi T, Aihara K. Transformation from complex networks to time series using classical multidimensional scaling In: Artificial Neural Networks–ICANN 2009. Springer; 2009 p. 325–334.

[30] Li X, Liu X, Tse CK. Recent advances in bridging time series and complex networks. In: Circuits and Systems (ISCAS), 2013 IEEE International Symposium on. IEEE; 2013. p. 2505–2508.

[31] Villafañe-Delgado M, Aviyente S. Transforming functional connectivity networks of the brain to signals based on the resistance distance. In: In press, Acoustics, Speech and Signal Processing (ICASSP), 2016 IEEE International Conference on. IEEE; 2016.

[32] Aviyente S, Mutlu AY. A time-frequency-based approach to phase and phase synchrony estimation. IEEE Transactions on Signal Processing. 2011;59(7):3086–3098. 10.1109/TSP.2011.2144589

[33] Leon C. Time-frequency analysis: theory and applications. USA: Pnentice Hall 1995;.

[34] Rihaczek A. Signal energy distribution in time and frequency. IEEE Transactions on information Theory. 1968;14(3):369–374. 10.1109/TIT.1968.1054157

[35] Lachaux JP, Rodriguez E, Martinerie J, Varela FJ. Measuring phase synchrony in brain signals. Human brain mapping. 1999;8(4):194–208. 10.1002/(SICI)1097-0193(1999)8:4<194::AID-HBM4>3.0.CO;2-C 10619414 PMC6873296

[36] Sporns O, Zwi JD. The small world of the cerebral cortex. Neuroinformatics. 2004;2(2):145–162. 10.1385/NI:2:2:145 15319512

[37] Stam CJ, Reijneveld JC. Graph theoretical analysis of complex networks in the brain. Nonlinear biomedical physics. 2007;1(1):1 10.1186/1753-4631-1-3 17908336 PMC1976403

[38] Onnela JP, Saramäki J, Kertész J, Kaski K. Intensity and coherence of motifs in weighted complex networks. Physical Review E. 2005;71(6):065103 10.1103/PhysRevE.71.065103 16089800

[39] Latora V, Marchiori M. Efficient behavior of small-world networks. Physical review letters. 2001;87(19):198701 10.1103/PhysRevLett.87.198701 11690461

[40] Watts DJ, Strogatz SH. Collective dynamics of ‘small-world’networks. nature. 1998;393(6684):440 10.1038/30918 9623998

[41] Humphries MD, Gurney K. Network ‘small-world-ness’: a quantitative method for determining canonical network equivalence. PloS one. 2008;3(4):e0002051 10.1371/journal.pone.0002051 18446219 PMC2323569

[42] Rossi F. Visualization methods for metric studies. In: Proceedings of the International Workshop on Webometrics, Informetrics and Scientometrics; 2006. p. 356–366.

[43] Hamon R, Borgnat P, Flandrin P, Robardet C. Nonnegative matrix factorization to find features in temporal networks. In: Acoustics, Speech and Signal Processing (ICASSP), 2014 IEEE International Conference on. IEEE; 2014. p. 1065–1069.

[44] Horvath S. Weighted network analysis: applications in genomics and systems biology. Springer Science & Business Media; 2011.

[45] Klein DJ, Randić M. Resistance distance. Journal of Mathematical Chemistry. 1993;12(1):81–95. 10.1007/BF01164627

[46] Ghosh A, Boyd S, Saberi A. Minimizing effective resistance of a graph. SIAM review. 2008;50(1):37–66. 10.1137/050645452

[47] Bapat R. Resistance matrix of a weighted graph. Communications in Mathematical and in Computer Chemistry/MATCH. 2004;50:73–82.

[48] Ellens W, Spieksma F, Van Mieghem P, Jamakovic A, Kooij R. Effective graph resistance. Linear algebra and its applications. 2011;435(10):2491–2506. 10.1016/j.laa.2011.02.024

[49] Bapat RB. Graphs and matrices. vol. 27 Springer; 2010.

[50] Villafañe-Delgado M. Assessment of Functional Connectivity in the Human Brain: Multivariate and Graph Signal Processing Methods. Signal Processing. 2016;64(11).

[51] Shannon CE, Weaver W, Burks AW. The mathematical theory of communication; 1951.

[52] Pearson K. X. Contributions to the mathematical theory of evolution.—II. Skew variation in homogeneous material. Philosophical Transactions of the Royal Society of London (A). 1895;(186):343–414. 10.1098/rsta.1895.0010

[53] Pearson K. “The Error Law and Its Generalizations By Fechner and Pearson.” A Rejoinder. Biometrika. 1905;4(1/2):169–212.

[54] Villafañe-Delgado M, Aviyente S. Graph information theoretic measures on functional connectivity networks based on graph-to-signal transform. In: Signal and Information Processing (GlobalSIP), 2016 IEEE Global Conference on. IEEE; 2016. p. 1137–1141.

[55] Hamon R, Borgnat P, Flandrin P, Robardet C. Duality between Temporal Networks and Signals: Extraction of the Temporal Network Structures. arXiv preprint arXiv:150503044. 2015;.

[56] Hamon R, Borgnat P, Flandrin P, Robardet C. From graphs to signals and back: Identification of network structures using spectral analysis. arXiv preprint arXiv:150204697. 2015;.

[57] Aicher C, Jacobs AZ, Clauset A. Learning latent block structure in weighted networks. Journal of Complex Networks. 2014;3(2):221–248. 10.1093/comnet/cnu026

[58] Moran TP, Bernat EM, Aviyente S, Schroder HS, Moser JS. Sending mixed signals: Worry is associated with enhanced initial error processing but reduced call for subsequent cognitive control. Social cognitive and affective neuroscience. 2015; p. nsv046. 10.1093/scan/nsv046 PMC4631152 25925270

[59] Eriksen BA, Eriksen CW. Effects of noise letters upon the identification of a target letter in a nonsearch task. Perception and psychophysics. 1974;16(1):143–149. 10.3758/BF03203267

[60] Kayser J, Tenke CE. Principal components analysis of Laplacian waveforms as a generic method for identifying ERP generator patterns: I. Evaluation with auditory oddball tasks. Clinical Neurophysiology. 2006;117(2):348–368. 10.1016/j.clinph.2005.08.034 16356767

[61] Cavanagh JF, Cohen MX, Allen JJB. Prelude to and resolution of an error: EEG phase synchrony reveals cognitive control dynamics during action monitoring. The Journal of Neuroscience. 2009;29(1):98–105. 10.1523/JNEUROSCI.4137-08.2009 19129388 PMC2742325

[62] Cohen MX. Error-related medial frontal theta activity predicts cingulate-related structural connectivity. Neuroimage. 2011;55(3):1373–1383. 10.1016/j.neuroimage.2010.12.072 21195774

[63] Hall JR, Bernat EM, Patrick CJ. Externalizing Psychopathology and the Error-Related Negativity. Psychological Science. 2007;18(4):326–333. 10.1111/j.1467-9280.2007.01899.x 17470258 PMC2242425

[64] Kotsiantis SB, Zaharakis ID, Pintelas PE. Machine learning: a review of classification and combining techniques. Artificial Intelligence Review. 2006;26(3):159–190. 10.1007/s10462-007-9052-3

[65] Fawcett T. ROC graphs: Notes and practical considerations for researchers. Machine learning. 2004;31(1):1–38.

[66] Dimitriadis SI, Antonakakis M, Simos P, Fletcher JM, Papanicolaou AC. Data-driven topological filtering based on orthogonal minimal spanning trees: application to multigroup magnetoencephalography resting-state connectivity. Brain connectivity. 2017;7(10):661–670. 10.1089/brain.2017.0512 28891322 PMC6435350

[67] Sohn H, Kim I, Lee W, Peterson BS, Hong H, Chae JH, et al Linear and non-linear EEG analysis of adolescents with attention-deficit/hyperactivity disorder during a cognitive task. Clinical Neurophysiology. 2010;121(11):1863–1870. 10.1016/j.clinph.2010.04.007 20659814

[68] Ozdemir A, Bolanos M, Bernat E, Aviyente S. Hierarchical spectral consensus clustering for group analysis of functional brain networks. IEEE Transactions on Biomedical Engineering. 2015;62(9):2158–2169. 10.1109/TBME.2015.2415733 25807564

